# Epidemiology of burn injuries in Nepal: a systemic review

**DOI:** 10.1186/s41038-017-0075-y

**Published:** 2017-04-03

**Authors:** Sanjib Tripathee, Surendra Jung Basnet

**Affiliations:** Plastic Surgery Department, Kirtipur Hospital, Kirtipur, Nepal

**Keywords:** Burns, Nepal, Epidemiology, Injury, Systemic review

## Abstract

Burn is a global public health problem associated with significant morbidity and mortality, mostly in low- and middle-income countries. Southeast-Asian countries share a big burden of burn injuries, and Nepal is not an exception. We performed a systemic review to examine the epidemiological characteristics of burn injures in Nepal. Relevant epidemiological studies were identified through systemic search in PubMed, EMBASE, and Google Scholar. Reference lists from relevant review articles were also searched. Studies were included if they meet our selection criteria. Eight studies were included in our systemic review. Most of the burn victims belong to the working age group between 15–60 years old. Flame burns were found to be the most common cause of burn injury followed by scald burns, whereas scald burns were the most common cause of burn injury among the pediatric population. Most patients sustained less severe burn injuries, with home being the most common place of burn injury. The average hospital stay among the burn victims ranged from 13 to 60 days. Mortality among the burn victims ranged from 4.5 to 23.5%, with highest mortality among the flame burn patients. Developed nations have significantly reduced the burn incidence through effective intervention program. Although, burn injuries are the leading cause of morbidity and mortality in Nepal, effective intervention programs are lacking due to the limited epidemiological data related to burn injuries. Further large scale research is imperative to investigate the problem and assess the effectiveness of an intervention program.

## Background

Burn injuries are a significant cause of morbidity and mortality throughout the world. Burn injuries perhaps represent the widest spectrum of any form of trauma. Burns occur in all age groups and may range in severity from very minor requiring no treatment to extremely severe requiring highest level of intensive treatment.

Burn is a public health problem, accounting for an estimated 265,000 deaths annually throughout the world [1]. The burden of burn is unevenly distributed throughout the world. The majority of these deaths occur in low- and middle-income countries (LMIC), and almost half occur in South-East Asia region including Nepal. Burns are among the leading causes of disability-adjusted life-years (DALYs) lost in LMIC. Burns are the third most common cause of injury in Nepal followed by fall injuries and road traffic accidents [2]. There is no factual report about the incidence of burn injuries in Nepal, but Nepal government estimates that there were 55,000 burn cases and 2100 deaths in 2008. Experts believe that the incidence of burn injuries and death rate are far more than the estimated figure. Lack of national burn registry makes this calculation very challenging. Five percent of disabilities at all age group in Nepal are due to burns related injury [3]. The high burden of burn injuries and lack of adequate epidemiological data makes it challenging for the policy makers to implement a proper strategic plan for burn prevention.

The epidemiological study is prerequisite for planning and implementing prevention program in the community. High income countries are able to reduce the burn incidence and mortality through proper epidemiological research and utilizing the information for planning preventive strategies. However, different strategic planning is required based on the cultural and socio-economic status of the region. The programs which are successful in developed countries might not be translated in developing countries. The purpose of this review is to identify the demographics, mechanism of injury, associated risk factors, and outcome of burn injury in Nepal. Being the first systemic review of burn injuries from Nepal, this information will be useful for policy makers and clinicians to further understand the epidemiological characteristics of burn injury in Nepal and also identify the research questions to be answered.

## Review

We conducted a comprehensive literature search of MEDLINE, EMBASE, and Google Scholar using the keywords “burns, epidemiology, Nepal, injury, flame burn, scald burn, electric burn, chemical burn, and contact burn”. Additional articles were identified by reviewing reference lists. The local journals were searched for the articles not indexed in PubMed or EMBASE. The inclusion criteria for the article were (1) articles which studied epidemiological characteristics of burn injury from Nepal, (2) only published articles were included in our study, and (3) only English language articles were included in our study. Articles were excluded if they fail to present the data for extraction, and also, the case studies were not included in our review.

The data were extracted from each study into an excel spreadsheet and further evaluation were done by two authors. The formal meta-analysis was not performed due to the large degree of clinical heterogeneity between the study populations. We followed the MOOSE guidelines while performing this review [4].

**Fig. 1:**
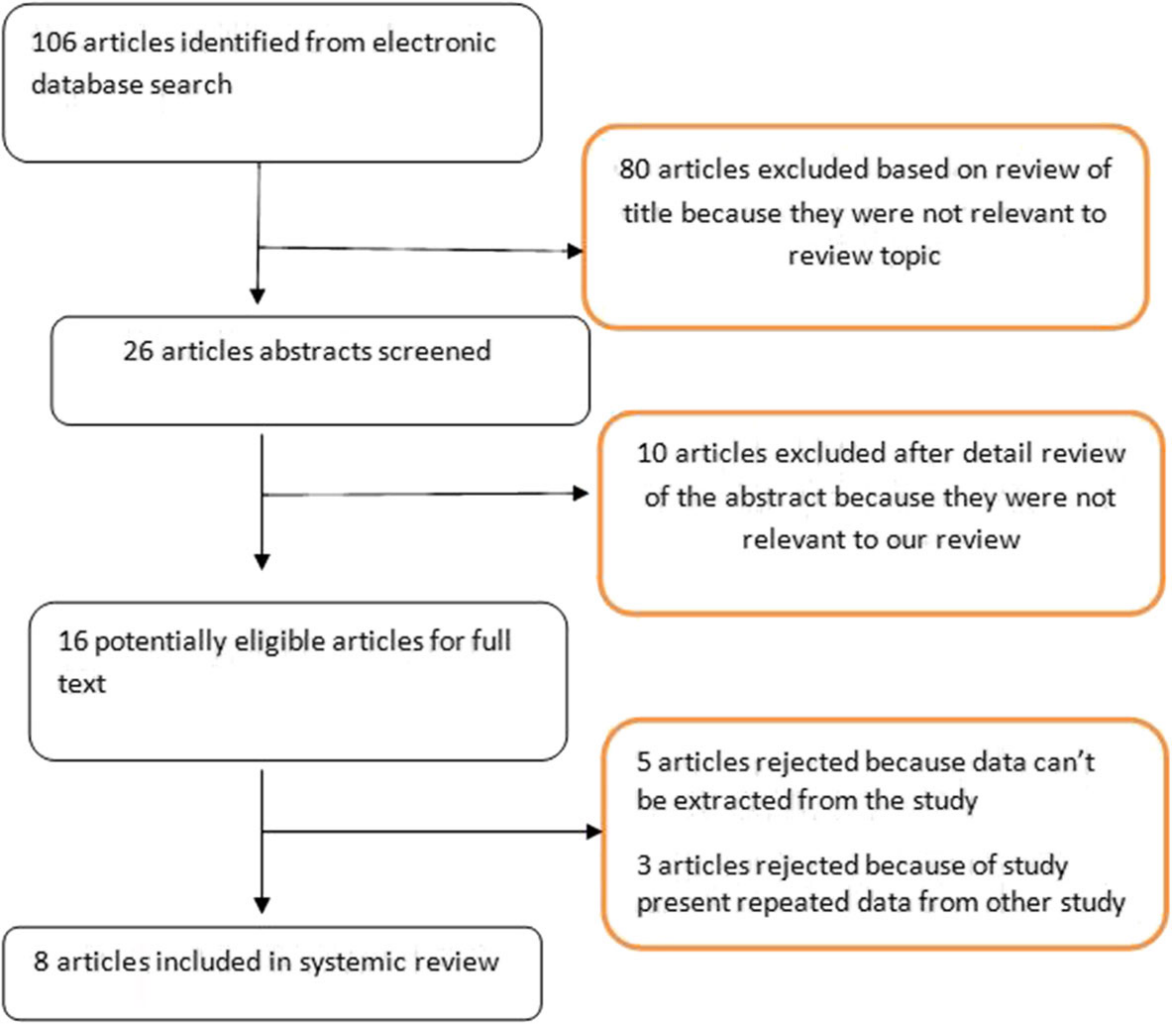
Selection process of articles for review

The database search elicited 106 articles. The process of article selection for systemic review is summarized in Fig.1.

### Results

The search revealed 106 titles of interests, of which eight were selected for the review. Out of eight articles included in our review, two were prospective cohort studies, four were retrospective cohort studies, and two were cross-sectional studies. Six studies were conducted in Kathmandu valley (capital), one study was from Pokhara, and one was generalized study from Nepal. These studies represent a total of 1710 burn patients over a period of 17 years (1998–2015). Eight studies included in our systemic review are shown in Table 1.

**Table 1:** Overview of the studies included in systemic review

Study	Year	Location	Study type	Age group	Total patient number﻿, n﻿	Male/female	Mortality, n(%)
Liu et al. [5]	1998	Pokhara	Prospective study	All age group	237	112/125	55(23.2)
Shrestha et al. [8]	2006	Kathmandu	Prospective study	Pediatric age group	22	10/12	1(4.5)
Poudel-Tandukar et al. [9]	2006	Kathmandu	Cross-sectional study	Middle school student	350	155/195	NA
Chalise et al. [10]	2008	Kathmandu	Retrospective study	All age group	50	29/21	7(14.0)
Dahal et al. [6]	2010	Kathmandu	Retrospective study	All age group	100	44/56	21(21.0)
Rai et al. [11]	2014	Kathmandu	Retrospective study	All age group	78	32/46	15(19.2)
Gupta et al. [29]	2015	Nationwide	Cross-sectional study	All age group	54	28/26	NA
Sharma et al. [7]	2015	Kathmandu	Retrospective study	All age group	819	428/391	168(20.5)

#### Demographics

Among eight studies in our review, five studies showed the predominance of female patients whereas three studies showed the predominance of male patients (Table 1); although there was no significant gender difference in any of these studies. Two studies which review the pediatric age group also showed the predominance of female patient. Study by Liu et al. [5] showed that burns were more common in pediatric age group which accounted for 61% in their study, whereas other five studies reported that burns were more common among the working age group population (15–59 years). Three studies by Liu et al. [5], Dahal et al. [6], and Sharma et al. [7] reported that mean age among the female burn patients was higher than the male patients (17.3 vs. 13.3 years, 34 vs. 26 years, and 35 vs. 28 years, respectively). Among the pediatric age group, burns among the children between 0–5 years were more common than the older children [8].

#### Mechanism of burn injury

The mechanism of burn injuries reported by individual studies is shown in Table 2. This table shows that majority of burns were caused by flame (five studies) and scald (three studies). Among the two studies on pediatric age group, both showed scald burns as the most common cause of burn injury and flame burn being the second most common cause of injury. Other studies also reported scald as the most common cause of burn injury among pediatric age group. One study reported electric burns as the second most common cause of injury [7]. Contact burns were reported by three studies, with highest proportion being 10.3% of all burns in pediatric patients [9]. Flame burn was the most common cause among the female patients whereas electric burns were common among the male patients.

**Table 2:** Mechanism of burn injury in Nepal

Study	Age group	Total patient number﻿, n﻿	Flame burnn n(%)	Scald burnn n(%)	Electric burn n n(%)	Contact burnn n(%)	Chemical burnn n(%)	Othersn n(%)
Liu et al. [5]	All ages	237	152(64.1)	67(28.3)	9(3.8)	1(0.4)	3(1.3)	5(2.1)
Shrestha et al. [8]	Pediatrics	22	10(45.4)	12(54.5)	–	–	–	–
Poudel-Tandukar et al. [9]	Pediatrics	350	125(35.7)	187(53.4)	–	36(10.3)	–	2(0.6)
Chalise et al. [10]	All ages	50	33(66.0)	8(16.0)	7(14.0)	–	2(4.0)	–
Dahal et al. [6]	All ages	100	64(64.0)	21(21.0)	14(14.0)	–	1(1.0)	–
Rai et al. [11]	All ages	78	48(61.5)	15(19.2)	11(14.1)	–	–	4(5.1)
Gupta et al. [29]	All ages	54	21(38.9)	32(59.3)	–	–	–	1(1.9)
Sharma et al. [7]	All ages	819	633(77.3)	69(8.4)	104(12.7)	2(0.2)	5(0.6)	6(0.7)

#### Place of burn injury

Most common place of burns was house according to the studies which reported the place of burn incident. Females were more likely to be injured in home, whereas males were most likely to be injured in workplace [7]. One study reported that 86% of childhood burn injuries occured inside the house [8].

#### Severity of burn injury

Five studies reported the severity of burn injuries based on percentage total body surface area (%TBSA) burn, ranging from 0.5 to 98%. Majority of patients sustained less severe burn injury. Mean body surface area was reported to be 16.3, 19.4, and 11% by three studies [5, 10, 11]. In pediatric burns, 96% of burn victim sustained less than 20% TBSA burn. Sharma et al. reported that burns sustained by female patients were significantly more than male patients (mean %TBSA 28% vs. 20%) [7].

#### Time escape till admission

Four studies reported the time escape between burn injury and hospital admission which ranged from 15 min to 48 days. Liu et al. reported that 31.8% of patients were admitted more than 1 week after injury whereas 2.5% admitted more than 6 weeks after injury [5]. Shrestha et al. reported 40% of pediatric patients were admitted within 12 h of injury [8].

#### Hospital stay

Six studies reported the hospital stay among the burn victims which ranged from 1 to 124 days. The average hospital stay among the burn victims ranged from 13 to 60 days. Sharma et al. reported that hospital stay among the female patients was significantly longer than male patients [7]. Shrestha et al. reported that in pediatric age group, 63% of patients were admitted for at least 20 days [8].

#### Mortality

Six studies reported mortality in their study population which ranged from 4.5 to 23.5%. Table 3 presents a detail of the studies. Most studies indicated that increased %TBSA burn as a significant predictor of mortality. Two studies showed that female mortality outnumbered male mortality [5, 7].

**Table 3:** Mortality among burn victims and its features

Study	Total patient number, n	Mortality, n(%)	Most common cause	Remarks
Liu et al. [5]	237	55(23.2)	Hypovolemia Sepsis	No patients with >40%TBSA survived Female to male death ratio 2.07
Shrestha et al. [8]	22	1(4.5)	Sepsis	–
Chalise et al. [10]	50	7(14.0)	–	%TBSA significant predicator of mortality
Dahal et al. [6]	100	21(21.0)	–	–
Rai et al. [11]	78	15(19.2)	–	Mortality proportional to increasing %TBSA burn. No patient with >40%TBSA survived
Sharma et al. [7]	819	168(20.5)	–	73% of females. Flame burn responsible for 95% deaths

TBSA Total burn surface area

### Discussion

This systemic review summarizes the epidemiological characteristics of burn injuries in Nepal as reported by 8 different studies, which includes 1710 patients over the period of 17 years. Although most studies were conducted in Kathmandu (Capital), the patients were represented from all over Nepal because the burn patients requiring surgery are referred to Kathmandu for further management from all over the country.

Burn injuries are among the leading causes of injury in Nepal [1]. Flame burns were the most common cause of burn injuries in Nepal, followed by scald burns. Two systemic reviews of epidemiology of burn injuries in South Asia and East Mediterranean region have also reported flame burn being the most common cause of burn injury [12, 13]. Flame burns were associated with considerably higher body surface area burn, and more females were found to sustain flame burns compared to males. In Nepalese culture, females are responsible for daily household activities like cooking. This is the reason that more females sustain flame burn compared to males. Furthermore, many household in Nepal use traditional open fire for cooking purpose which further increase the risk of burn injuries [14]. Among the pediatric patients, scald burns were the most common cause of burn injury. Other studies also reported scalds as the most common cause of burn injury among the pediatric population [15–18]. Although the mortality rate is low in pediatric group, they are more susceptible for developing disability caused by burn injuries. Study by Sharma et al. reported electric burns as the second most common cause of burn injury, this might be caused because their study consists of fewer pediatric population who are more susceptible for scald burns [7]. One study in Bangladesh found that 2% of pediatric burn victims were permanently disabled, estimating a disability rate of 5.7 per 100,000 children [19]. The study from Nepal reported that 5% of disability in all age group is due to burn injury [3].

Although house is considered to be a safe place, most burn injuries occurred inside the house itself, mostly occurring in kitchen. The use of open fire for cooking, wearing loose fitting cloths like saree, and poorly regulated LPG (liquefied petroleum gas) cylinders increase the risk of burn injuries in the Nepalese population. Our review indicates that time escape between burn injury and initiation of proper burn treatment significantly increases the morbidity and mortality among the burn victims. Nepal is a mountainous country where many parts are not linked by transportation, many burn victims from these regions could never make to hospital, and even if they make to hospital, they are presented very late complicating the treatment. In Nepal, limited number of burn center and trained physician make it difficult for burn victims to get proper treatment immediately after the injury. Burn patients in most centers are managed by general physician or general surgeon. Patient lost their golden hour for treatment being referred from one hospital to another hospital for burn management.

Mortality of burn victims in Nepal is comparable to findings from other LMIC [20–22].The highest mortality rate in our study was 23.5%, and the lowest was 4.5% [5, 8].The high mortality rate in Liu et al. study might be caused due to various reasons like the flame burn was the most common cause of burn injury which tends to burn more body surface area, 31.8% of patients presented more than 1 week after injury, and lack of well-trained physician and resources for burn treatment at the center [5]. On the other hand, low burn mortality of 4.5% in the study by Shrestha et al. might be due to the fact that this study was conducted in pediatric population where scald burns were the most common cause of injury which tends to be less severe [8]. Furthermore, most of the patients were brought to hospital within 24 h after injury. The mortality rate in developed country is very low compared to LMIC [23–25].The factors contributing to high mortality in Nepal compared to developed nations are lack of proper first-aid measures, time lost between the injury, and initiation of proper treatment, poor economic status, and malnutrition among the general population.

Female patients and increased %TBSA were significant risk factor for the mortality in different studies [5, 7, 10, 11]. More females sustained flame burns compared to male patients, and flame burns were associated with increased %TBSA burn, which may be a reason for higher mortality among the female patient compared to males. Other studies from LMICs also coincide with the finding that mortality increases with increasing %TBSA [13, 26, 27]. The mortality rate for patient with more than 40%TBSA burns almost approaches 100% in Nepal. This is unimaginable in developed countries, but it is a normal scenario in most of the developing countries around the world. Most burn patients in Nepal are from low socio-economic status unable to afford the expensive burn treatment. Furthermore, lack of skin substitute to cover the excised burn wound makes the treatment more challenging in these settings. Additionally, prevalence of malnutrition among these populations further worsens the situation [28].

#### Strengths and limitations of the study

This review of the epidemiological characteristics of burn injury in Nepal is the first systemic review from Nepal which provides an analysis of the trends in burn injury. We used a standard systemic review methodology including selection of studies and data extraction. There was considerable heterogeneity between the studies, which limit the comparison of some finding reported by the studies. Although we conducted thorough search of literature on PubMed, EMBASE, and Google scholar and also complete our search by reviewing related and cross-referencing literature, existence of missing studies can never be excluded. The majority of data were obtained from the hospitals, limiting their generalization on a population level.

## Conclusions

Burn injuries are a public health problem in Nepal, which is also one of the leading causes of injury-related morbidity and mortality. Although Nepal shares a big burden of burn injury, the studies concerning burn epidemiology and management are limited. To our best knowledge, this article is the first systemic review that provides an overview of epidemiological characteristic of burn injury from Nepal. We believe this article further helps clinicians and policymaker understand the epidemiological characteristics of burn injuries in Nepal and aids in implementing effective preventive strategies. Most cases of burn injuries are preventable, and we believe that if we can educate the people about burn injury and its preventive strategy, it will help reduce the burn morbidity and mortality to the great extent. Government and clinicians should focus on the preventive strategy rather than just treating the acute burn patients.

## References

[1] WHO Media Center Fact Sheets: Burns [Internet]. 2014 [cited 2016/05/04]. Available from: http://www.who.int/mediacentre/factsheets/fs365/en/.

[2] Gupta S, Wong EG, Nepal S, Shrestha S, Kushner AL, Nwomeh BC (2015). Injury prevalence and causality in developing nations: results from a countrywide population-based survey in Nepal. Surgery.

[3] NB T. Report of injury survey in Bhumisthan Village Panchayat Dhadhing and in Bir Hospital Kathmandu. Stockholm: National Programmes on Accident and Injury Prevention National Board of Health and Welfare, Stockholm; 1989.

[4] Stroup DF, Berlin JA, Morton SC, Olkin I, Williamson GD, Rennie D (2000). Meta-analysis of observational studies in epidemiology: a proposal for reporting. Jama.

[5] Liu EH, Khatri B, Shakya YM, Richard BM (1998). A 3 year prospective audit of burns patients treated at the Western Regional Hospital of Nepal. Burns.

[6] Dahal P PB. Pattern of Burn patients admitted in a Burn Unit of Bir Hospital Kathmandu. Post Graduate Med J Natl Acad Med Sci. 2010;10(02):62–4.

[7] Sharma NP, Duke JM, Lama BB, Thapa B, Dahal P, Bariya ND (2015). Descriptive epidemiology of unintentional burn injuries admitted to a tertiary-level government hospital in Nepal: gender-specific patterns. Asia Pac J Public Health.

[8] Shrestha SR (2006). Burn injuries in pediatric population. JNMA.

[9] Poudel-Tandukar K, Nakahara S, Ichikawa M, Poudel KC, Joshi AB, Wakai S. Unintentional injuries among school adolescents in Kathmandu, Nepal: a descriptive study. Public Health. 2006;120(7):641–9. 10.1016/j.puhe.2006.01.012 16759678

[10] Chalise PR, Shrestha S, Sherpa K, Nepal U, Bhattachan CL, Bhattacharya SK (2008). Epidemiological and bacteriological profile of burn patients at Nepal Medical College Teaching Hospital. Nepal Med Coll J.

[11] Rai SM, Karki B, Nakarmi K, Ghartimagar M, Nagarkoti K, Joshi KD (2014). Retrospective study on early outcome of acute burn injuries treated at Nepal Cleft and Burn Centre of Public Health Concern Trust-Nepal. J Nepal Health Res Counc.

[12] Golshan A, Patel C, Hyder AA (2013). A systematic review of the epidemiology of unintentional burn injuries in South Asia. J Public Health (Oxf).

[13] Othman N, Kendrick D (2010). Epidemiology of burn injuries in the East Mediterranean Region: a systematic review. BMC Public Health.

[14] Rhodes EL, Dreibelbis R, Klasen E, Naithani N, Baliddawa J, Menya D (2014). Behavioral attitudes and preferences in cooking practices with traditional open-fire stoves in Peru, Nepal, and Kenya: implications for improved cookstove interventions. Int J Environ Res Public Health.

[15] Alaghehbandan R, MacKay Rossignol A, Rastegar LA (2001). Pediatric burn injuries in Tehran, Iran. Burns.

[16] Maghsoudi H, Samnia N (2005). Etiology and outcome of pediatric burns in Tabriz, Iran. Burns.

[17] Oztorun CI, Demir S, Azili MN, Senayli A, Livanelioglu Z, Senel E (2016). The outcomes of becoming a pediatric burn center in Turkey. Ulus Travma Acil Cerrahi Derg.

[18] Lee CJ, Mahendraraj K, Houng A, Marano M, Petrone S, Lee R (2016). Pediatric burns: a single institution retrospective review of incidence, etiology, and outcomes in 2273 burn patients (1995–2013). J Burn care Res.

[19] Mashreky SR, Rahman A, Chowdhury SM, Giashuddin S, SvanstrOm L, Linnan M (2008). Epidemiology of childhood burn: yield of largest community based injury survey in Bangladesh. Burns.

[20] Maghsoudi H, Pourzand A, Azarmir G (2005). Etiology and outcome of burns in Tabriz, Iran. An analysis of 2963 cases. Scand J Surg.

[21] Calder F (2002). Four years of burn injuries in a Red Cross hospital in Afghanistan. Burns.

[22] Mukerji G, Chamania S, Patidar GP, Gupta S (2001). Epidemiology of paediatric burns in Indore, India. Burns.

[23] Santos JV, Oliveira A, Costa-Pereira A, Amarante J, Freitas A (2016). Burden of burns in Portugal, 2000–2013: a clinical and economic analysis of 26,447 hospitalisations. Burns.

[24] Song C, Chua A (2005). Epidemiology of burn injuries in Singapore from 1997 to 2003. Burns.

[25] Burton KR, Sharma VK, Harrop R, Lindsay R (2009). A population-based study of the epidemiology of acute adult burn injuries in the Calgary Health Region and factors associated with mortality and hospital length of stay from 1995 to 2004. Burns.

[26] Forjuoh SN (2006). Burns in low- and middle-income countries: a review of available literature on descriptive epidemiology, risk factors, treatment, and prevention. Burns.

[27] Potokar TS, Prowse S, Whitaker IS, Ali S, Chamania S (2008). A global overview of burns research highlights the need for forming networks with the developing world. Burns.

[28] Gurung G (2010). Social determinants of protein-energy malnutrition: need to attack the causes of the causes. J Health Popul Nutr.

[29] Gupta S, Mahmood U, Gurung S, Shrestha S, Kushner AL, Nwomeh BC (2015). Burns in Nepal: a population based national assessment. Burns.

