# Erratum to: Epidemiology of burn injuries in Nepal: a systemic review

**DOI:** 10.1186/s41038-017-0081-0

**Published:** 2017-05-24

**Authors:** Sanjib Tripathee, Surendra Jung Basnet

**Affiliations:** Plastic Surgery Department, Kirtipur Hospital, Kirtipur, Nepal

## Erratum

After the publication of this article [1] the editor noticed some erroneous ‘n’ values in the sub-headings of Table 2. The original article has been updated to omit these values. The correct updated version of the table is also shown below.

**Table 2:** Mechanism of burn injury in Nepal

Study	Age group	Total patient number, n	Flame burn (%)	Scald burn (%)	Electric burn (%)	Contact burn (%)	Chemical burn (%)	Others (%)
Liu et al. [5]	All ages	237	152(64.1)	67(28.3)	9(3.8)	1(0.4)	3(1.3)	5(2.1)
Shrestha et al. [8]	Pediatrics	22	10(45.4)	12(54.5)	–	–	–	–
Poudel-Tandukar et al. [9]	Pediatrics	350	125(35.7)	187(53.4)	–	36(10.3)	–	2(0.6)
Chalise et al. [10]	All ages	50	33(66.0)	8(16.0)	7(14.0)	–	2(4.0)	–
Dahal et al. [6]	All ages	100	64(64.0)	21(21.0)	14(14.0)	–	1(1.0)	–
Rai et al. [11]	All ages	78	48(61.5)	15(19.2)	11(14.1)	–	–	4(5.1)
Gupta et al. [29]	All ages	54	21(38.9)	32(59.3)	–	–	–	1(1.9)
Sharma et al. [7]	All ages	819	633(77.3)	69(8.4)	104(12.7)	2(0.2)	5(0.6)	6(0.7)
